# Risk factors associated with meningitis outbreak in the Upper West Region of Ghana: A matched case-control study

**DOI:** 10.1371/journal.pone.0305416

**Published:** 2024-08-26

**Authors:** Moses Musah Kabanunye, Benjamin Noble Adjei, Daniel Gyaase, Emmanuel Kweku Nakua, Stephen Opoku Afriyie, Yeetey Enuameh, Michael Owusu

**Affiliations:** 1 Department of Epidemiology and Biostatistics, School of Public Health, Kwame Nkrumah University of Science and Technology, Kumasi, Ghana; 2 Centre for Health System Strengthening, Kumasi, Ghana; 3 Department of Medical Diagnostics, Faculty of Allied Health Sciences, Kwame Nkrumah University of Science and Technology, Kumasi, Ghana; Public Health England, UNITED KINGDOM

## Abstract

The Northern part of Ghana lies within the African meningitis belt and has historically been experiencing seasonal meningitis outbreaks. Despite the continuous meningitis outbreak in the region, the risk factors contributing to the occurrence of these outbreaks have not been clearly identified. This study, therefore, sought to describe the clinical characteristics and possible risk factors associated with meningitis outbreaks in the Upper West Region (UWR). A 1:2 matched case-control study was conducted in May-December 2021 to retrospectively investigate possible risk factors for meningitis outbreak in the UWR of Ghana between January and December 2020. Cases were persons with laboratory confirmed meningitis, and controls were persons of similar age and sex without meningitis living in the same house or neighborhood with a confirmed case. Both primary and secondary data including clinical, socio-demographic and laboratory information were collected and entered on standard questionnaires. Data was analyzed using descriptive statistics and conditional logistic regression. Meningitis cases were mostly due to *Streptococcus pneumoniae* (67/98; 68.37%), followed by *Neisseria meningitides* serogroup X (27/98; 27.55%). Fever occurred in 94.03% (63/67) of *Streptococcus pneumoniae* cases and 100% in both *Neisseria meningitidis* serogroup X (27/27) and *Neisseria meningitidis* serogroup W groups (3/3). CSF white cell count was significantly associated with the causative agents of meningitis. Conditional logistic regression analysis showed that, passive exposure to tobacco [AOR = 3.65, 95%CI = 1.03–12.96], bedrooms with 3 or more people [AOR = 4.70, 95%CI = 1.48–14.89] and persons with sore throat infection [AOR = 8.97, 95%CI = 2.73–29.43] were independent risk factors for meningitis infection. Headache, fever and neck pain continue to be the most common symptoms reported by meningitis patients. Education and other preventive interventions targeting exposure to tobacco smoke and crowded rooms would be helpful in reducing meningitis outbreaks in the Upper West Region of Ghana.

## Introduction

Bacterial meningitis remains a global health concern with a high number of adverse clinical outcomes despite the use of effective conjugate vaccines against most bacterial pathogens. According to the WHO, an estimated 2.82 million cases of meningitis are recorded every year across the globe, with over 300,000 deaths [1]. A third of these cases occur among children less than 6 years [1]. Globally, the incidence rate of bacterial meningitis varies by region, and this variation is mainly due to environmental-related factors, including climate conditions and socioeconomic disparities. A study done in the United States, Europe, South America, and Australia reported that the annual rate of meningococcal meningitis ranged from 0.12 to 3 cases per 100,000 population [2].

The northern region of Ghana falls within the meningitis belt in Africa, as outlined by WHO [1]. The area has been grappling with seasonal meningitis outbreaks for several years, particularly during the dry-hot season, despite concerted efforts from governments, organizations, individuals, and health experts [3–5]. In response to these outbreaks, Ghana introduced the meningococcal conjugate vaccine (MenAfriVac^®^) in the three northern regions (Upper East, Northern and Upper West) and included a 13-valent pneumococcal conjugate vaccine (PCV13) into its routine infant national immunization program in 2012, according to the recommendations of WHO [6, 7].

These interventions generally led to a reduction in cases of meningitis, however recent reports indicate the occurrence of meningitis outbreaks involving serotype 1 pneumococcal meningitis [8]. This calls for a holistic approach at evaluating the entire chain of transmission. In a 5-year retrospective study in Ghana’s meningitis belt, 395 cases were confirmed to be *Neisseria meningitides*, and *Neisseria meningitides serogroup X* was found to be most prevalent, with 43% of cases from the Upper West Region [9]. Between 2010 and 2015, there were 4,561 documented cases of meningitis, resulting in the tragic loss of over 400 lives, according to the Ghana Health Service [10].

The situation escalated in March 2020 with the onset of the COVID-19 outbreak in Ghana, burdening the region with a dual challenge of both COVID-19 and meningitis outbreaks. During this period more than 400 cerebrospinal meningitis (CSM) cases, with about 50 deaths were reported in the country’s CSM endemic regions: Northern, North East, Savanna, Upper East and Upper West Regions. [10–13].

The fear and uncertainty stemming from the COVID-19 pandemic caused a shift in focus among local and national health entities away from meningitis prevention and control measures [11].

The persistent recurrence of these outbreaks emphasize the need for a broader approach at addressing meningitis outbreaks in Ghana. One important focus is to identify the risk factors and clinical characteristics associated with these outbreaks in order to improve the index of suspicion inform policy directions in providing interventions. This study, therefore, describes the clinical characteristics and risk factors associated with meningitis outbreaks in the Upper West region of Ghana. Findings from this study will provide evidence that will inform Public Health Officials about the specific socio-demographic and clinical characteristics associated with meningitis infection so that prevention activities can be tailored to them to avert future outbreaks and associated effects in the UWR.

## Materials and methods

### Study design and population

The study employed a matched case-control design (1:2 cases to control) to explore the risk factors associated with the meningitis outbreak in the Upper West Region of Ghana between January and December 2020. The case-control study design is appropriate to build evidence of an association between risk factors and meningitis outbreak.

### Study setting

The region has eleven administrative districts. There are eight District Hospitals and one Regional Hospital in the region. Most of the inhabitants are farmers. Per the 2021 population and housing census, the Upper West Region has a population of 901,502 of which 51.2% are females. Also, 73.6% of the people reside in rural areas. The major economic activities in the region are farming, and trading.

#### Study population

A ***case*** (meningitis) in this study was defined as a person confirmed to have meningitis through laboratory testing between January and December 2020. A person was deemed positive for meningitis if a microbial pathogen was identified from the cerebrospinal fluid either by PCR or culture or Gram’s stain.

***Controls*** were defined as persons of similar age and sex without the disease (meningitis) living in the same house or neighborhood with a confirmed case between January and December 2020.

#### Inclusion and exclusion criteria

The study included persons who met the following criteria:

a. Persons with laboratory-confirmed meningitis.
b. Persons similar in age and sex living in the same house or neighbourhood as a laboratory-confirmed case at the time they were identified.

The following cases were excluded in the study

a. Laboratory-confirmed cases who died
b. Individuals who did not consent to the study
c. Individuals who could not be reached

#### Selection of cases

**Fig 1:**
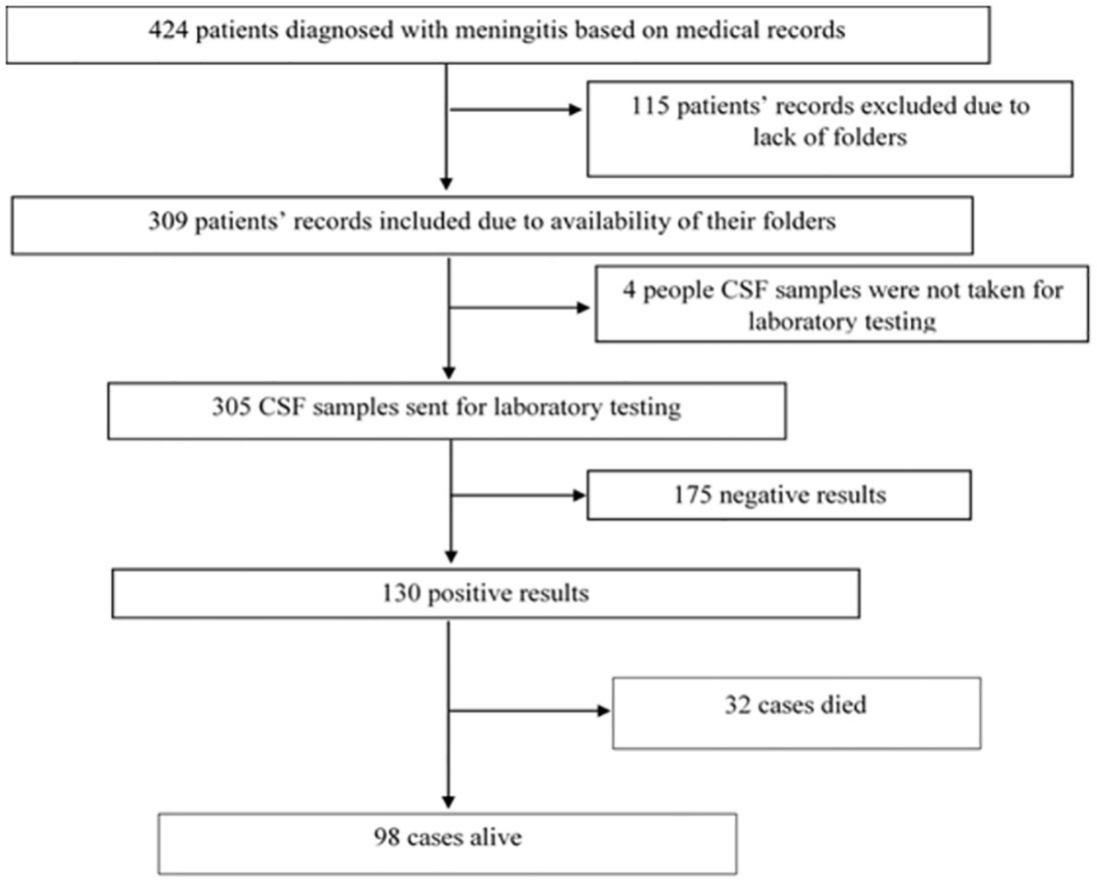
Flow chart of record cases selected.

In order to select cases, a line list of all suspected meningitis cases were obtained from the Regional Surveillance Office. Information obtained from the line list was used to obtain the case-based forms from the various Health Directorates and Public Health Units of the Hospitals. From the list, a total of 424 suspected cases were identified. The case-based forms of these suspected cases were used to obtain information on their folder numbers. The folder numbers were used to retrieve their folders from the records department of the various treatment centers. Of the 424 suspected cases, only the folders for 309 were found. All the retrieved folders were reviewed and data on socio-demographic and clinical characteristics, and laboratory results were extracted. After the records review of the 309 folders, a total of 305 had laboratory results of which 4 did not. Of the 305 results, 130 were laboratory-confirmed positive while 175 were negative results. Of the 130 laboratory confirmed cases of meningitis, 32 died and 98 survived. To assess the risk factors for the meningitis outbreak, the 32 confirmed cases who died were excluded, and controls were not assigned to them. Fig 1 is a flow chart showing how the 98 cases were selected.

#### Selection of controls

*Controls were selected by locating the addresses of the households of cases*. Addresses of the 98 confirmed cases who survived were retrieved from their folders or case-based forms. The addresses were used to trace them to their homes. In the homes of the cases, persons who had similar characteristics (age and sex) were purposively selected as controls. When eligible controls were not available in the same house, the next three to four houses around were explored, and controls were selected from there.

### Sample size determination

A total of 294 individuals consisting of 98 cases and 196 controls were enrolled in the study. The census method was used in selecting the cases for this study. As such, all the cases (n = 98) that survived and were alive at the time of the study were enrolled in the study. Two controls were purposively selected for each of the 98 surviving cases. The selection of two controls per case was done in order to increase the power of the study [14].

### Data collection

Both primary and secondary sources of data were used in the study. Secondary data were obtained from laboratory records and primary data through structured paper-based questionnaire.

A structured questionnaire was used to collect primary data from both cases and controls. The questionnaire contained information on possible risk factors of the meningitis outbreak, including their demographic characteristics (age, sex, occupation etc.). The respondents were interviewed face-to-face using local languages. In a situation where the study participant was a child, a caretaker or a responsible adult within the same household who had adequate knowledge of the child’s activities was interviewed. A data extraction form was developed after reviewing relevant literature. This form was then used to extract data through records review of the folders of the confirmed meningitis cases. Some of the extracted data included socio-demographic characteristics, clinical, and laboratory findings of the confirmed cases.

### Statistical analysis

All statistical analyses were carried out using STATA Version 16 (StataCorp, College Station, TX, USA). Descriptive statistics were performed to describe the variables under study. Means and their corresponding standard deviations were estimated for normally continuous distributed variables. Medians and interquartile range (IQR) were also estimated for skewed continuous variables. In addition, frequencies and percentages were calculated for categorical variables. Kruskal-Wallis equality-of-populations rank testwas used to identify differences between groups in continuous outcomes (laboratory findings) and causative agents. A bivariate and multivariable conditional logistic regression model was used to explore the potential risk factors of the meningitis outbreak. Variables that had a probability value of 0.2 or less at the univariate level were included in the multivariable analyses. Variables that had a p-value less than 0.05 at 95% confidence interval in the multivariable analyses were considered statistically significant.

#### Key definitions

Previous sore throat infection in this study was defined as anyone who suffered from an upper respiratory tract infection at most two weeks prior to the development of signs and symptoms of meningitis and the subsequent diagnosis of the disease in the person.

Neighborhood in this study was defined as the larger compound in which the case lives or the house or structure next to the structure of the case.

Exposure to tobacco smoke was defined as persons who smoked themselves (first-hand smokers) or were exposed to tobacco smoke as a result of the association with smokers (second-hand smokers).

Contact with a case per this study was defined as persons who live and interact with a confirmed meningitis case in their neighbourhood.

### Ethical consideration

Approval for the study was obtained from the Committee on Human Research and Publication Ethics, Kwame Nkrumah University of Science and Technology (CHRPE/AP/148/21). Written informed consent forms were made available to participants who were 18 years or above before enrolment in the study. Child assent and parental/guardian consent were obtained for children less than 18 years. The anonymity and confidentiality of the participants were assured using unique codes. Participants who did not provide consent were excluded from the study.

## Results

### Socio-demographic characteristics of cases and controls

Table 1 presents the demographic characteristics of both cases and controls. A total of 98 cases of meningitis and 196 controls were investigated. The mean age (standard deviation) of the cases was 24.7 (±20.9) years and that of the controls was 24.6 (±20.5) years. Majority of the cases (51.02%) were less than age 15 years and the least (10.23%) were those in the age groups 30–44 and above 60 years. About 68.37% of the participants (cases and controls) were males while 32.00% were females. There were no statistically significant differences between the cases and controls for their socio-demographic characteristics (p-value > 0.05).

**Table 1:** Socio-demographic characteristics of participants by cases and controls.

Variable	Cases		Controls		All		χ^2^	p value
	n = 98	%	n = 196	%	n = 294	%		
**Age (years)**							0.21	0.99
Mean ±SD	24.7±20.9		24.6±20.5		24.6±20.6			
<15	50	51.02	99	50.51	149	50.68		
15–29	16	16.33	30	15.31	46	15.65		
30–44	10	10.20	23	11.73	33	11.22		
45–59	12	12.24	25	12.76	37	12.59		
60+	10	10.20	19	9.69	29	9.86		
**Sex**							0.07	0.79
Female	31	31.63	62	31.63	93	31.63		
Male	67	68.37	134	68.37	201	68.37		
**Occupation**							2.01	0.85
Artisan	7	7.14	21	10.71	28	9.52		
Employed	6	6.12	7	3.57	13	4.42		
Farming	13	13.27	28	14.29	41	13.95		
Student	49	50.00	98	50.00	147	50.00		
Trader	9	9.18	16	8.16	25	8.50		
Unemployed	14	14.29	26	13.27	40	13.61		
**Religion**							3.05	0.22
Christianity	74	75.51	138	70.41	212	72.11		
Islam	19	19.39	53	27.04	72	24.49		
Traditionalist	5	5.10	5	2.55	10	3.40		
**Marital status**							0.42	0.81
Single	60	61.22	120	61.22	180	61.22		
Married	33	33.67	69	35.20	102	34.69		
Divorced/widowed	5	5.10	7	3.57	12	4.08		
**Educational level**							1.71	0.64
No education	29	29.59	53	27.04	82	27.89		
Basic	50	51.02	107	54.59	157	53.40		
Secondary	12	12.24	28	14.29	40	13.61		
Tertiary	7	7.14	8	4.08	15	5.10		

### Clinical characteristics of meningitis cases caused by different bacteria

A total of 98 confirmed cases of meningitis were identified and included in this study. Of this, 67 (68.37%) were due to *Streptococcus pneumoniae*, 27 (27.55%) were *Neisseria meningitidis* serogroup X cases and 3 (3.06%) of the cases were due to *Neisseria meningitidis* serogroup W. The most common triad of symptoms reported were fever, headache and neck pains. Fever was present in about 94.03% *Streptococcus pneumoniae* cases and 100% in both *Neisseria meningitidis* serogroup X *and Neisseria meningitidis* serogroup W groups. Of all the meningitis cases recorded, only 7.46% of the *Streptococcus pneumonia cases*, 33.33% of *Neisseria meningitidis* serogroup W and 18.52% of *Neisseria meningitidis* serogroup X cases had been vaccinated with menA against meningitis. Table 2 describes the clinical characteristics of meningitis cases according to causative agents.

**Table 2:** Clinical characteristics of meningitis cases according to causative agents.

Clinical presentation	*Streptococcus pneumoniae* n = 67 (%)	*Haemophilus influenzae* type b n = 1 (%)	*Neisseria meningitidis* serogroup W n = 3 (%)	*Neisseria meningitidis* serogroup X n = 27 (%)	Total n = 98 (%)
**Glasgow coma score at presentation**					
Severe score (3–8)	6 (12.0)	0 (0.00)	0 (0.00)	0 (0.00)	6 (8.45)
Moderate score (9–14)	7 (14.0)	0 (0.00)	1 (33.33)	1 (5.88)	9 (12.68)
Normal Score (15)	37 (74.00)	1 (100.00)	2 (66.67)	16 (94.12)	56 (78.87)
Vaccinated with MenA	5 (7.46)	0 (0.00)	1 (33.33)	5 (18.52)	11 (11.22)
Had fever	63 (94.03)	0 (0.00)	3 (100.00)	27 (100.00)	93 (94.90)
Headache	60 (89.55)	1 (100.00)	3 (100.00)	23 (85.19)	87 (88.78)
Neck pains	47 (70.15)	0 (0.00)	3 (100.00)	17 (67.96)	67 (68.37)
Nausea/Vomiting	26 (38.81)	0 (0.00)	1 (33.33)	11 (40.74)	38 (38.78)
Photophobia	23 (34.33)	0 (0.00)	2 (66.67)	6 (22.22)	31 (31.63)
Petechial rash	5 (7.46)	0 (0.00)	0 (0.00)	3 (11.11)	8 (8.16)
Pre-hospital seizure	4 (5.97)	0 (0.00)	0 (0.00)	3 (11.11)	7 (7.14)
Seizure during hospitalization	5 (7.46)	0 (0.00)	0 (0.00)	2 (7.41)	7 (7.14)
Shock	1 (1.49)	0 (0.00)	0 (0.00)	0 (0.00)	1 (1.02)
Coma	2 (2.99)	0 (0.00)	0 (0.00)	0 (0.00)	2 (2.04)
Positive Kernig’s sign	41 (61.19)	0 (0.00)	1 (33.33)	13 (48.15)	55 (56.12)
Positive Brudzinki’s sign	32 (47.76)	0 (0.00)	1 (33.33)	14 (51.85)	47 (47.96)

### Clinical characterization of CSF among meningitis cases

Evaluation of the laboratory results showed the average white blood cell count was 12.00 (IQR = 7.50–15.00) cells per x10^9^/l with the highest white blood cell count found in *Neisseria meningitidis* serogroup X group (median = 15.00, IQR = 6.97–19.00 μl). Most of the appearance of the CSF was cloudy (76.53%), followed by clear (17.35%) while 3.06% of the CSF samples were bloody and xanthochromic in appearance. The highest CSF white cell count was in the *Neisseria meningitidis* serogroup W group (median = 30.00, IQR = 8.00–30.00 μl) and the lowest CSF white cells count was seen in *Haemophilus influenza* type 2 group (4.00, IQR = 4.00–4.00 μl). Table 3 below presents laboratory findings of the cases according to their pathogens.

**Table 3:** Laboratory findings of meningitis cases according to causative agents.

Laboratory finding	*Streptococcus pneumoniae* n = 67	*Haemophilus influenzae* type b n = 1	*Neisseria meningitidis* serogroup W n = 3	*Neisseria meningitidis* serogroup X n = 27	Total n = 98	p-value
Blood WBC x10^9^/l	12.15 (7.8–15.0)	9 (9.0–9.0)	12.03 (6.97–19.0)	15.00 (6.97–19.00)	12 (7.5–15.0)	0.760
**HB level, g/dl (n = 92)**	12 (11.2–12.8)	9.5 (9.50–9.50)	10.8 (10.8–11.7)	11.8 (10.7–12.8)	12 (10.85–12.75)	0.050
**CSF colour, n (%)**						0.684
Clear	10 (14.93)	1 (100.0)	0 (0.00)	6 (22.22)	17 (17.35)	
Cloudy	53 (79.10)	0 (0.00)	3 (100.0)	19 (70.37)	75 (76.53)	
Bloody	2 (2.99)	0 (0.00)	0 (0.00)	1 (3.70)	3 (3.06)	
Xanthochromic	2 (2.99)	0 (0.00)	0 (0.00)	1 (3.70)	3 (3.06)	
CSF WCC (per μl)	12.56 (5.0–12.0)	4.0 (4.0–4.0)	30.0 (8.0–30.0)	10.0 (10.0–20.0)	10.0 (5.0–15.0)	0.045
CSF glucose count (mmol/l)	2.60 (1.19–3.00)	2.90 (2.90–2.90)	0.24 (0.04–0.44)	2.0 (0.13–3.00)	2.60 (1.1–3.0)	0.130
CSF protein count (g/l)	3.0 (2.5–4.2)	0.0 (0.0–0.0)	3.43 (0.17–6.70)	2.40 (1.60–4.00)	3.0 (1.6–4.2)	0.798

WBC = white blood cell, CSF = Cerebrospinal fluid, IQR = Interquartile range, g/l = gram per litre, mmol/L = millimol per litre, μl = micro litre and WCC = white cell count, g/dl = gram per deciliter,

### Risk factors for meningitis outbreak

Table 4 below presents the descriptive characteristics of the possible risk factors associated with meningitis outbreak in the Upper West Region. The majority of the cases (59.18%) were passive smokers while 81.12% of the controls never smoked. A little over eighty (80.61%) percent of the cases lived in rooms that housed at least three persons compared to 72.96% of the controls stayed in rooms that contains less than 3 people. For contact with a meningitis case, 48.98% of the cases came into contact with confirmed meningitis case compared to 12.24% of the controls. Majority of both cases and controls never use a urinary catheter (78.57% and 92.86% respectively).

**Table 4:** Descriptive characteristics of risk factors by cases and controls.

Variable	Cases		Controls		All		COR	p-value
	n = 98	%	n = 196	%	n = 294	%		
**Age (years)**								
Mean ±SD	24.7±20.9		24.6±20.5		24.6±20.6			
**Sex**								
Female	31	31.63	62	31.63	93	31.63		
Male	67	68.37	134	68.37	201	68.37		
**Exposure to tobacco Smoke**								
Never smoked	36	36.73	159	81.12	195	66.33	1	
Passive smoker	58	59.18	36	18.37	94	31.97	6.85	0.001
Active smoker	4	4.08	1	0.51	5	1.70	18.93	0.014
**Indoor kitchen**								
No	19	19.39	75	38.27	94	31.97	1	
Yes	79	80.61	121	61.73	200	68.03	3.01	0.001
**Number of windows**								
None	40	40.82	16	8.16	56	19.05	1	
One	48	48.98	68	34.69	116	39.46	0.13	0.001
Two	10	10.20	112	57.14	122	41.50	0.01	0.001
**Number of persons in room**								
Less than 3	19	19.39	143	72.96	162	55.10	1	
3 or more	79	80.61	53	27.04	132	44.90	10.08	0.001
**Contact with a case**								
No	50	51.02	172	87.76	222	75.51	1	
Yes	48	48.98	72	12.24	72	24.49	7.79	0.001
**Previous sore throat infection**								
No	16	16.33	164	83.67	180	61.22	1	
Yes	82	83.67	32	16.33	114	38.78	21.60	0.001
**On urinary catheter**								
No	87	88.78	194	98.98	281	95.58	1	
Yes	11	11.22	2	1.02	13	4.42	20.54	0.004
**Previous surgery**								
No	77	78.57	182	92.86	259	88.10	1	
Yes	21	21.43	14	7.14	35	11.90	4.65	0.001

COR = Crude Odds Ratio

Table 5 below presents the conditional logistic regression analysis of the possible risk factors associated with the meningitis outbreak in the UWR of Ghana. The odds of having meningitis were 6.85 and 18.93 times higher in passive and active tobacco smokers [COR = 6.85, 95%CI = 3.71–12.67; COR = 18.93, 95%CI = 1.80–199.37] respectively compared to those who never smoked tobacco. Having 3 or more persons in a bedroom had ten times higher odds of having meningitis compared to less than 3 persons in a bedroom [COR = 10.08, 95%CI = 5.15–19.73].

**Table 5:** Factors associated with meningitis cases.

Variable	Unadjusted			Adjusted			
	COR	p-value	95%CI	AOR	p-value	95%CI	
**Exposure to tobacco Smoke**							
Never smoked	1			1			
Passive smoker	6.85	**0.001**	3.71–12.67	3.65	**0.045**		1.03–12.96
Active smoker	18.93	**0.014**	1.80–199.37	0.42	0.578		0.02–8.74
**Indoor kitchen**							
No	1			1			
Yes	3.01	**0.001**	1.60–5.67	4.07	0.084		0.83–19.95
**Number of windows**							
None	1			1			
One	0.13	**0.001**	0.04–0.36	0.12	**0.046**		0.02–0.96
Two	0.01	**0.001**	0.00–0.039	0.03	**0.005**		0.00–0.34
**Number of persons in room**							
Less than 3	1			1			
3 or More	10.08	**0.001**	5.15–19.73	4.70	**0.009**		1.48–14.89
**Contact with a case**							
**No**	1			1			
**Yes**	7.79	**0.001**	3.90–15.55	1.47	0.563		0.40–5.38
**Previous sore throat infection**							
No	1			1			
Yes	21.60	**0.001**	9.38–49.76	8.97	**0.001**		2.73–29.43
**On urinary catheter**							
No	1			1			
Yes	20.54	**0.004**	2.64–159.75	3.55	0.448		0.14–93.16
**Previous surgery**							
No	1			1			
Yes	4.65	**0.001**	1.94–11.13	0.86	0.887		0.11–6.67

COR = crude odds ratio; AOR = adjusted odds ratio

In the multivariable conditional logistic regression analysis, it was revealed that after accounting for the effect of other variables, a person who had exposure to tobacco smoke (passive tobacco smokers/second-hand tobacco smokers) had 3.65 times higher odds of contracting meningitis compared to a person who never smoked (AOR = 3.65, 95%CI = 1.03–12.96). The odds of contracting meningitis were 88% lower in persons living in bedrooms with one window and 97% lower in persons living in bedrooms with two windows compared to living in a bedroom without a window [AOR = 0.12, 95%CI = 0.02–0.96, AOR = 0.03, 95%CI = 0.00–0.34] respectively. Also, the odds of contracting meningitis were 4.70 times higher in bedrooms with 3 or more persons compared to bedrooms with less than 3 persons [AOR = 4.70, 95%CI = 1.48–14.89].

## Discussion

In this study, fever, headache and neck stiffness were the three main complaints reported by the cases. Similar studies conducted in Lithuania, the Netherlands and Ethiopia all found fever and neck pains as the commonly complained symptoms among meningitis patients [15–17]. Fever was present in all cases due to *Neisseria meningitidis* serogroup X (100%) and *Neisseria meningitidis* serogroup W (100%) and also in 94.03% of the cases due to *Streptococcus pneumoniae*. This suggests that the tendency of fever to occur in infected meningitis patients may contributed by presence of aetiological agents. However, further studies are required to better understand the dependence of fever on the causative agents of meningitis in infected patients.

Most of the CSF samples analyzed were cloudy in appearance. A longitudinal study conducted in Ethiopia supports the current study finding, as they also reported 61.2% of 85 CSF samples analyzed were turbid while the remaining 38.8% were clear in appearance [18, 19]. The cloudy CSF indicates the presence of white or red blood cells, microbes, or an increase in protein levels. Therefore, this could serve as a surrogate marker to determine the positivity of meningitis in cloudy CSF samples. Persons whose CSF are cloudy must therefore be screened for bacterial meningitis.

This study found a link between passive exposure to tobacco smoke and an increased risk of contracting meningitis infection in the Upper West Region of Ghana. Previous studies also reported the relationship between passive exposure to tobacco smoke with *H*. *influenzae* and pneumococcal meningitis [20, 21]. Passive smoking is suggested to lower the body’s immune system [20, 21], and this could negatively affect the body’s defense against infections including meningitis. This increased risk of meningococcal infection in passive smokers could be attributed to a higher risk of contracting the meningococcal pathogen through contact with smokers rather than a direct effect of passive smoke. However, the mechanism by which passive exposure to tobacco smoke increases the risk of meningitis infection is still an assumption. Further studies are needed to fully unravel the truth in this belief.

We also found a higher risk of meningitis acquisition amongst individuals who reported a sore throat infection in the dry season prior to contracting meningitis. Upper respiratory tract infections and meningococcal carriage have been reported in the past [22, 23]. Upper respiratory tract infection, together with dust and low humidity, could be necessary drivers for the high-risk meningitis outbreak in the dry season. This assumption is reinforced by a previous study conducted in Burkina Faso, which also found an association between upper respiratory tract infections and an outbreak of meningitis [24, 25]. Sore throat infection could likely be due to the effects of the dry cold and dusty winds blowing, and that could have led to the occurrence of cracks and sores in the respiratory tract, thus creating an easy entryway for the meningococcal pathogens and subsequently leading to a meningitis infection. This could also be attributed to a coexisting unrelated infection that predisposes a person to contract meningitis.

Additionally, this study found the presence of at least one window in a bedroom to be protective against meningitis compared to bedrooms without a window. The presence of window(s) improves ventilation and allows for free circulation of air. This helps blow any potential disease-causing pathogen away that may have been trapped in these rooms by the wind. Most of the bedrooms in the study area, through observation, were clustered and without windows. Some of the rooms had small windows but were covered with aluminum sheets, or in the event, it was not covered, it was a small opening. This, therefore, makes the free circulation of air in such rooms obstructed, making them fertile grounds for the spread of meningitis.

Again, the presence of a meningitis case in a compound increases the risk of other household members to contracting the same infection due to frequent contact. The presence of a case in the same bedroom frequents the contact with other household members. In the study area, many people still practice the extended family system, mostly in the rural areas. Due to this, a lot of people still sleep in the same room at night. In the event where one of them contracts a contagious disease (meningitis), the risk of spreading it among the other roommates is increased. A previous study conducted also found an increased risk between meningitis infection and the presence of a case in the house [26].

## Conclusion

This study has identified exposure to tobacco smoke, bedrooms with 3 or more persons staying in them, and persons with sore throat infection prior to the outbreak of meningitis as important risk factors of meningitis in the Upper West Region. Bedrooms that had at least a window were found to be associated with a lower risk of acquiring meningitis. The study recommends an improved laboratory surveillance to help detect all possible cases of meningitis that are missed by clinicians and public health professionals in the study area. There is also a need for education on the use of tobacco, early recognition of symptoms and improvement in building design to reduce the risk of meningitis in the Upper West region.

## Supporting information

S1 Checklist Clinical studies checklist. (DOCX)

S2 Checklist STROBE checklist. (DOCX)

S1 Data Dataset for study: DOI 10.17605/OSF.IO/5F32X. (XLSX)
