# Supplementary material for: Risk factors associated with meningitis outbreak in the Upper West Region of Ghana: A matched case-control study
Source: PLoS One. 2024 Aug 26;19(8):e0305416. doi: 10.1371/journal.pone.0305416 (PMC11346653; doi:10.1371/journal.pone.0305416)
Supplement: S2 Checklist — (DOCX) [file pone.0305416.s002.docx]

STROBE Statement—checklist of items that should be included in reports of observational studies

|  | Item No. | Recommendation | Page  No. | Relevant text from manuscript |
| --- | --- | --- | --- | --- |
| **Title and abstract** | 1 | (*a*) Indicate the study’s design with a commonly used term in the title or the abstract | 1 | Risk factors associated with meningitis outbreak in North-Western Ghana: A matched case-control study. |
|  |  | (*b*) Provide in the abstract an informative and balanced summary of what was done and what was found | 2 | A 1:2 matched case-control study was conducted in May-December 2021 to retrospectively investigate possible risk factors for meningitis outbreak in the Upper West Region of Ghana between January and December 2020.  Meningitis cases were mostly due to *Streptococcus pneumoniae* (68.37%), followed by *Neisseria meningitides* serotype X (27.55%). Fever occurred in 94.03% of *Streptococcus pneumoniae* cases and 100% in both *Neisseria meningitidis* serotype X and *Neisseria meningitidis* serotype W groups  Conditional logistic regression analysis showed that, passive exposure to tobacco [AOR=3.65, 95%CI=1.03-12.96], bedrooms with 3 or more people [AOR=4.70, 95%CI=1.48-14.89] and persons with sore throat infection [AOR=8.97, 95%CI=2.73-29.43] were independent risk factors for meningitis infection |
| Introduction | | | |  |
| Background/rationale | 2 | Explain the scientific background and rationale for the investigation being reported | 3 | The Northern part of Ghana lies within the African meningitis belt and has historically been experiencing seasonal meningitis outbreaks. Despite the continuous meningitis outbreak in the region, the risk factors contributing to the occurrence of these outbreaks have not been clearly identified. Findings from this study will provide evidence that will help Public Health Officials to know the specific socio-demographic and clinical characteristics that are associated with meningitis infection so that prevention activities can be tailored to them to avert future outbreaks and associated effects. |
| Objectives | 3 | State specific objectives, including any prespecified hypotheses | 3 | The study, therefore, sought to describe the clinical characteristics and risk factors associated with meningitis outbreaks in the North-Western part of Ghana. |
| Methods | | | |  |
| Study design | 4 | Present key elements of study design early in the paper | 5 | A matched case-control study (1:2 cases to control) was conducted to examine the risk factors for meningitis outbreak in the North-Western part of Ghana. |
| Setting | 5 | Describe the setting, locations, and relevant dates, including periods of recruitment, exposure, follow-up, and data collection | 4 | This study was conducted at the Upper West Region of Ghana between May and December 2021. The region has eleven administrative districts. There are eight District Hospitals and one Regional Hospital in the region. Most of the inhabitants are farmers. A case (meningitis) in this study was defined as a person confirmed to have meningitis through laboratory testing between January and December 2020. Controls were defined as persons of similar age and sex without the disease (meningitis) living in the same house or neighborhood with a confirmed case between January and December 2020. |
| Participants | 6 | (*a*) *Cohort study*—Give the eligibility criteria, and the sources and methods of selection of participants. Describe methods of follow-up  *Case-control study*—Give the eligibility criteria, and the sources and methods of case ascertainment and control selection. Give the rationale for the choice of cases and controls  *Cross-sectional study*—Give the eligibility criteria, and the sources and methods of selection of participants | 5 | A case (meningitis) in this study was defined as a person confirmed to have meningitis through laboratory testing between January and December 2020. A person was deemed positive for meningitis if a microbial pathogen was identified from the cerebrospinal fluid either by PCR or culture or Gram’s stain.  Controls were defined as persons of similar age and sex without the disease (meningitis) living in the same house or neighborhood with a confirmed case between January and December 2020. |
|  |  | (*b*) *Cohort study*—For matched studies, give matching criteria and number of exposed and unexposed  *Case-control study*—For matched studies, give matching criteria and the number of controls per case | 5 | A total of 98 alive cases of meningitis were recruited and included in this study. Two controls were purposively selected for each of the 98 surviving cases. In all, 196 controls were selected for the 98 surviving cases to explore the potential risk factors of the meningitis outbreak. |
| Variables | 7 | Clearly define all outcomes, exposures, predictors, potential confounders, and effect modifiers. Give diagnostic criteria, if applicable | 5 | A person was deemed positive for meningitis if a microbial pathogen was identified from the cerebrospinal fluid either by PCR or culture or Gram’s stain. More details shown in Figure 2 |
| Data sources/ measurement | 8* | For each variable of interest, give sources of data and details of methods of assessment (measurement). Describe comparability of assessment methods if there is more than one group | 5, 6 | A total of 424 medical records of cases of meningitis that occurred within the study period were reviewed. A structured questionnaire was used to collect primary data from both cases and controls. The questionnaire contained information on possible risk factors of the meningitis outbreak, including their demographic characteristics (age, sex, occupation etc.). The respondents were interviewed face-to-face using local languages. In a situation where the study participant was a child, a caretaker or a responsible adult within the same household who had adequate knowledge of the child’s activities was interviewed. |
| Bias | 9 | Describe any efforts to address potential sources of bias | 5 | Participants who met the eligibility criteria were randomly selected. Figure 2 shows how selection of cases was done |
| Study size | 10 | Explain how the study size was arrived at | 5, 6 | A total of 294 individuals consisting of 98 cases and 196 controls were enrolled in the study. The census method was used in selecting the cases for this study. As such, all the cases (n=98) that survived and were alive at the time of the study were enrolled in the study. Two controls were purposively selected for each of the 98 surviving cases. |

Continued on next page

| Quantitative variables | 11 | Explain how quantitative variables were handled in the analyses. If applicable, describe which groupings were chosen and why | 6 | Descriptive statistics were performed to describe the variables under study. Means and their corresponding standard deviations were estimated for normally continuous distributed variables. Medians and interquartile range (IQR) were also estimated for skewed continuous variables. |
| --- | --- | --- | --- | --- |
| Statistical methods | 12 | (*a*) Describe all statistical methods, including those used to control for confounding | 6 | Shown in Statistical Analysis section of the manuscript |
|  |  | (*b*) Describe any methods used to examine subgroups and interactions | 6 | Kruskal-Wallis equality-of-populations rank test; a nonparametric test was used to identify differences between groups in continuous outcomes (laboratory findings) and causative agents. A bivariate and multivariable conditional logistic regression model was used to explore the potential risk factors of the meningitis outbreak. Variables that had a probability value of 0.2 or less at the univariate level were included in the multivariable analyses. |
|  |  | (*c*) Explain how missing data were addressed | 5 | The records were screened for completeness of clinical information and availability of laboratory results. Those with missing details were excluded from analysis as shown in Fig 2. |
|  |  | (*d*) *Cohort study*—If applicable, explain how loss to follow-up was addressed  *Case-control study*—If applicable, explain how matching of cases and controls was addressed  *Cross-sectional study*—If applicable, describe analytical methods taking account of sampling strategy | 5 | A total of 98 alive cases of meningitis were recruited and included in this study. Two controls were purposively selected for each of the 98 surviving cases. A case (meningitis) in this study was defined as a person confirmed to have meningitis through laboratory testing between January and December 2020. A person was deemed positive for meningitis if a microbial pathogen was identified from the cerebrospinal fluid either by PCR or culture or Gram’s stain.  Controls were defined as persons of similar age and sex without the disease (meningitis) living in the same house or neighborhood with a confirmed case between January and December 2020. |
|  |  | (*e*) Describe any sensitivity analyses | Not applicable | Not applicable |
| Results | | | | |
| Participants | 13* | (a) Report numbers of individuals at each stage of study—eg numbers potentially eligible, examined for eligibility, confirmed eligible, included in the study, completing follow-up, and analysed | 5, 7 | Shown in Fig 2 and Table 1 |
|  |  | (b) Give reasons for non-participation at each stage | 5 | Shown in Fig 1 |
|  |  | (c) Consider use of a flow diagram | 5 | Shown in Fig 1 |
| Descriptive data | 14* | (a) Give characteristics of study participants (eg demographic, clinical, social) and information on exposures and potential confounders | 8, 9, 10 | Shown in Tables 1, 2, 3 |
|  |  | (b) Indicate number of participants with missing data for each variable of interest | Does not apply | Does not apply |
|  |  | (c) *Cohort study*—Summarise follow-up time (eg, average and total amount) | Not applicable | Not applicable |
| Outcome data | 15* | *Cohort study*—Report numbers of outcome events or summary measures over time | Not applicable | Not applicable |
|  |  | *Case-control study—*Report numbers in each exposure category, or summary measures of exposure | 7, 12 | Shown in Tables 1 and 4 |
|  |  | *Cross-sectional study—*Report numbers of outcome events or summary measures | Does not apply | Does not apply |
| Main results | 16 | (*a*) Give unadjusted estimates and, if applicable, confounder-adjusted estimates and their precision (eg, 95% confidence interval). Make clear which confounders were adjusted for and why they were included | 11, 12 | Shown in Table 4 |
|  |  | (*b*) Report category boundaries when continuous variables were categorized | 9, 10 | Shown in Table 3 |
|  |  | (*c*) If relevant, consider translating estimates of relative risk into absolute risk for a meaningful time period | Does not apply | Does not apply |

Continued on next page

| Other analyses | 17 | Report other analyses done—eg analyses of subgroups and interactions, and sensitivity analyses | 8, 9 | Clinical characteristics of cases according to causative agents have been shown in Table 2. Moreover, Table 3 provides insight into other laboratory findings of cases |
| --- | --- | --- | --- | --- |
| Discussion | | | | |
| Key results | 18 | Summarise key results with reference to study objectives | 12, 13, 14 | In this study, fever, headache and neck stiffness were the three topmost complaints reported by the cases.  This study found a link between passive exposure to tobacco smoke and an increased risk of contracting meningitis infection in the North-Western Region of Ghana.  Also, this matched case-control study found a higher risk of meningitis acquisition amongst individuals who reported a sore throat infection in the dry season prior to contracting meningitis.  Additionally, this study found the presence of at least one window in a bedroom to be protective against meningitis compared to bedrooms without a window.  Again, the presence of a meningitis case in a compound increases the risk of other household members to contracting the same infection due to frequent contact. |
| Limitations | 19 | Discuss limitations of the study, taking into account sources of potential bias or imprecision. Discuss both direction and magnitude of any potential bias | None specified | None specified |
| Interpretation | 20 | Give a cautious overall interpretation of results considering objectives, limitations, multiplicity of analyses, results from similar studies, and other relevant evidence | 15 | This study has identified exposure to tobacco smoke, bedrooms with 3 or more persons staying in them, and persons with sore throat infection prior to the outbreak of meningitis as important risk factors of meningitis. Bedrooms that had at least a window were found to be associated with a lower risk of acquiring meningitis. |
| Generalisability | 21 | Discuss the generalisability (external validity) of the study results | 15 | This study has identified exposure to tobacco smoke, bedrooms with 3 or more persons staying in them, and persons with sore throat infection prior to the outbreak of meningitis as important risk factors of meningitis. Bedrooms that had at least a window were found to be associated with a lower risk of acquiring meningitis. The study recommends an improved laboratory surveillance to help detect all possible cases of meningitis that are missed by clinicians and public health professionals. There is also a need for education on the use of tobacco, early recognition of symptoms and improvement in building design to reduce the risk of meningitis. |
| Other information | |  | | |
| Funding | 22 | Give the source of funding and the role of the funders for the present study and, if applicable, for the original study on which the present article is based | 15 | The authors received no specific funding for this work. |

*Give information separately for cases and controls in case-control studies and, if applicable, for exposed and unexposed groups in cohort and cross-sectional studies.

**Note:** An Explanation and Elaboration article discusses each checklist item and gives methodological background and published examples of transparent reporting. The STROBE checklist is best used in conjunction with this article (freely available on the Web sites of PLoS Medicine at http://www.plosmedicine.org/, Annals of Internal Medicine at http://www.annals.org/, and Epidemiology at http://www.epidem.com/). Information on the STROBE Initiative is available at www.strobe-statement.org.
